# Quantification of prevalence, clinical characteristics, co-existence, and geographic variations of traditional Chinese medicine diagnostic patterns via latent tree analysis-based differentiation rules among functional dyspepsia patients

**DOI:** 10.1186/s13020-022-00656-x

**Published:** 2022-08-30

**Authors:** Leonard Ho, Yulong Xu, Nevin L. Zhang, Fai Fai Ho, Irene X. Y. Wu, Shuijiao Chen, Xiaowei Liu, Charlene H. L. Wong, Jessica Y. L. Ching, Pui Kuan Cheong, Wing Fai Yeung, Justin C. Y. Wu, Vincent C. H. Chung

**Affiliations:** 1 School of Chinese Medicine, Faculty of Medicine, The Chinese University of Hong Kong, Hong Kong, Hong Kong; 2 School of Information Technology, Henan University of Chinese Medicine, Zhengzhou, Henan China; 3 Department of Computer Science and Engineering, School of Engineering, The Hong Kong University of Science and Technology, Hong Kong, Hong Kong; 4 Xiangya School of Public Health, Central South University, 5/F, 238 Shang-Ma-Yuan-Ling Alley, Kai-Fu District, Changsha, Hunan China; 5 Department of Gastroenterology, Xiangya Hospital, Changsha, Hunan China; 6 Hunan International Scientific and Technological Cooperation Base of Artificial Intelligence Computer-Aided Diagnosis and Treatment for Digestive Disease, Changsha, Hunan China; 7 The Jockey Club School of Public Health and Primary Care, Faculty of Medicine, The Chinese University of Hong Kong, Hong Kong, Hong Kong; 8 Institute of Digestive Diseases, The Chinese University of Hong Kong, Hong Kong, Hong Kong; 9 School of Nursing, Faculty of Health and Social Sciences, The Hong Kong Polytechnic University, Hong Kong, Hong Kong; 10 Department of Medicine and Therapeutics, Faculty of Medicine, The Chinese University of Hong Kong, Hong Kong, Hong Kong

**Keywords:** Medicine, Chinese traditional, Diagnosis, Dyspepsia, Machine learning, Cluster analysis

## Abstract

**Background:**

Traditional Chinese Medicine (TCM) treatment strategies are guided by pattern differentiation, as documented in the eleventh edition of the International Classification of Diseases (ICD). However, no standards for pattern differentiation are proposed to ensure inter-rater agreement. Without standardisation, research on associations between TCM diagnostic patterns, clinical features, and geographical characteristics is also not feasible. This diagnostic cross-sectional study aimed to (i) establish the pattern differentiation rules of functional dyspepsia (FD) using latent tree analysis (LTA); (ii) compare the prevalence of diagnostic patterns in Hong Kong and Hunan; (iii) discover the co-existence of diagnostic patterns; and (iv) reveal the associations between diagnostic patterns and FD common comorbidities.

**Methods:**

A total of 250 and 150 participants with FD consecutively sampled in Hong Kong and Hunan, respectively, completed a questionnaire on TCM clinical features. LTA was performed to reveal TCM diagnostic patterns of FD and derive relevant pattern differentiation rules. Multivariate regression analyses were performed to quantify correlations between different diagnostic patterns and between diagnostic patterns and clinical and geographical variables.

**Results:**

At least one TCM diagnostic pattern was differentiated in 70.7%, 73.6%, and 64.0% of the participants in the overall (*n* = 400), Hong Kong (*n* = 250), and Hunan (*n* = 150) samples, respectively, using the eight pattern differentiation rules derived. 52.7% to 59.6% of the participants were diagnosed with two or more diagnostic patterns. Cold-heat complex (59.8%) and spleen-stomach dampness-heat (77.1%) were the most prevalent diagnostic patterns in Hong Kong and Hunan, respectively. Spleen-stomach deficiency cold was highly likely to co-exist with spleen-stomach qi deficiency (adjusted odds ratio (AOR): 53.23; 95% confidence interval (CI): 21.77 to 130.16). Participants with severe anxiety tended to have liver qi invading the stomach (AOR: 1.20; 95% CI: 1.08 to 1.33).

**Conclusions:**

Future updates of the ICD, textbooks, and guidelines should emphasise the importance of clinical and geographical variations in TCM diagnosis. Location-specific pattern differentiation rules should be derived from local data using LTA. In future, patients’ pattern differentiation results, local prevalence of TCM diagnostic patterns, and corresponding TCM treatment choices should be accessible to practitioners on online clinical decision support systems to streamline service delivery.

**Supplementary Information:**

The online version contains supplementary material available at 10.1186/s13020-022-00656-x.

## Introduction

### Background

Functional dyspepsia (FD) is a disorder of gut-brain interaction characterised by postprandial fullness, early satiety, epigastric burning, and/or epigastric pain [1]. These symptoms are unexplainable by routine gastrointestinal examinations such as oesophagogastroduodenoscopy and Helicobacter pylori (*H.* *pylori*) test [1]. Patients with predominant postprandial fullness or early satiety are categorised into the symptom subtype of postprandial distress syndrome (PDS) [2], while those with predominant epigastric burning or epigastric pain into epigastric pain syndrome (EPS) [2]. An overlapping of PDS and EPS may also occur [2].

Regardless of disease definitions, FD is estimated to be affecting 10–40% of the population in Western countries and 5–30% in Asia [3]. Unfortunately, as the first-line conventional treatments for FD in North America [4], proton pump inhibitors have limited effectiveness [5], with potentially serious adverse effects followed by long-term usage [4]. Prokinetics, the first-line conventional treatment common prescribed in Asia [6], have limited effect on symptomatic improvement [7]. Despite potential positive effects [8], some patients tend to avoid tricyclic antidepressants due to the perceived stigma of receiving psychiatric therapy [9].

The symptoms of FD resemble those of stomach pain and stomach stuffiness and fullness in Traditional Chinese Medicine (TCM) [10]. Herbal medicine is recommended by the latest Asian clinical guideline for FD [6], and its effectiveness has been supported by meta-analyses [11, 12]. One of the major differences between TCM and conventional medicine is that treatment strategies are guided by pattern differentiation in TCM practice. Pattern differentiation refers to the overall analysis of a patient’s clinical features to determine the disease’s location, cause, and nature [13–15]. Although the World Health Organization has endorsed pattern differentiation through a dedicated supplementary chapter in the eleventh edition of the International Classification of Diseases [16], standardised rules for the pattern differentiation process have not been proposed for any medical conditions. Without such standardisation, the TCM diagnostic process is likely to have a low inter-rater agreement [17], leading to substantial variations in diagnostic-to-treatment decisions and hence the quality of care. The incorporation of pattern differentiation into TCM clinical research is also hindered by the lack of standardised TCM diagnostic instruments. However, variations in TCM diagnostic patterns across different clinical and geographical characteristics may exist as per traditional theory, and thus the use of a single standardised diagnostic instrument may not always be appropriate. Using a fictitious TCM practice scenario presented below, we aimed to provide a clinically relevant context for explaining the importance and expected benefits of standardising TCM pattern differentiation for FD, as well as for other diseases.

#### Clinical problem 1: standardising traditional Chinese medicine pattern differentiation

A team of senior TCM practitioners in Hong Kong specialising in gastroenterology would like to standardise TCM diagnosis of FD for the department in order to reduce inter-rater variability in the diagnostic-to-treatment decision-making process among practitioners. They might first search for TCM clinical textbooks or guidelines that summarise TCM experts’ opinions to inform the standardisation process. Nevertheless, the TCM diagnostic schemes mentioned in these clinical textbooks are usually described in a “one-size-fits-all” manner without considering clinical relevance [18]. For instance, a diagnostic study showed that menopausal women in London, United Kingdom, presented with a wide variety of TCM diagnostic patterns, such as liver yin deficiency, phlegm-fluid retention, and qi stagnation and blood stasis in the chest [19]. This contrasted with the diagnostic schemes mentioned in TCM gynaecology textbooks, which only describe the TCM diagnostic patterns of kidney yang deficiency or kidney yin deficiency [19, 20]. The impracticality of this “one-size-fits-all” approach was also reflected by the fact that a wide variety of Chinese herbal formulae were prescribed in China for menopausal women, and most of these formulae were not addressing kidney yang deficiency or kidney yin deficiency as described in the clinical textbooks [18]. The same problem is prevalent among TCM clinical guidelines and has partly contributed to poor guideline adherence among TCM practitioners in China [21].

Given the limited clinical relevance of TCM clinical textbooks and guidelines, the senior TCM practitioners decided to search for TCM diagnostic instruments supported by research evidence instead. Such instruments might guide the collection of patients’ TCM clinical feature data (i.e*.*, signs and symptoms) in a repeatable manner, which might then facilitate standardisation [22, 23]. However, a systematic review revealed that the existing TCM diagnostic instruments for FD were of poor quality in terms of their development process and measurement properties [22]. The insufficient quality development process indicated that the instrument items were unlikely to be clinically relevant for measuring TCM diagnostic patterns, and the items might be poorly understood by patients [24]. With low-quality measurement properties, these instruments were prone to errors originating from poor reliability and validity [24]. In light of the current dearth of useable diagnostic instruments, de novo development of evidence-based pattern differentiation rules with appropriate quantitative approaches, such as latent tree analysis (LTA) [25–27] with data collected from diagnostic cross-sectional studies, is warranted [23].

LTA can be used to develop score-based pattern differentiation rules for TCM diagnoses [25–27]. Each rule is presented with a numerical score for each of the constituting clinical features of that TCM diagnostic pattern, as well as a threshold, for pattern differentiation [25–27]. If the total score of a patient exceeded that threshold, that patient would be classified as having that TCM diagnostic pattern and vice versa [25–27]. With such pattern differentiation rules, the senior TCM practitioners could reliably assess the prevalence of different TCM diagnostic patterns of FD in Hong Kong. If a particular TCM diagnostic pattern were found to be highly prevalent, the Chinese herbal treatment(s) addressing that pattern with high pre-test probability would be a common choice for many patients, informing treatment decision-making among TCM practitioners. There is a need to assess whether this approach can be applied to most FD patients.

#### Clinical problem 2: co-existing traditional Chinese medicine diagnoses among individual patients

Diagnosing co-existing TCM diagnostic patterns in an individual patient might also be of high interest to practitioners. In routine practice, it is not uncommon to differentiate more than one TCM diagnostic pattern in a patient, yet this is often not explicitly mentioned in TCM textbooks [13, 28]. A diagnostic cross-sectional study illustrated that 63.3% of sampled FD patients presented with two co-existing TCM diagnostic patterns, and 38.8% had at least three [23]. The TCM expert consensus on FD management in China [10] advised that TCM practitioners should expect to encounter a co-existence of two or more TCM diagnostic patterns. Accordingly, treatment strategies should target the dominant pattern [10], but quantitative rules for operationalising this approach are not yet available.

To address this problem, the practitioners could conduct diagnostic cross-sectional studies using the LTA-derived rules and quantify the prevalence of different TCM diagnostic patterns among FD patients. Regression analyses could then be performed to quantify the correlations between those TCM diagnostic patterns. These findings would inform TCM practitioners on the pre-test probabilities of different combinations of co-occurring patterns, facilitating the tailoring of Chinese herbal medicine formulae for managing complex needs.

#### Clinical problem 3: traditional Chinese medicine diagnostic patterns and comorbidities

Evidence from epidemiological studies showed a higher prevalence of depression [29, 30] and anxiety [30] among FD patients than in healthy individuals. These psychiatric comorbidities were hypothesised to contribute to the worsening of dyspeptic symptoms via disruption of the gut-brain axis [31–33]. FD patients with more severe dyspeptic symptoms indeed have a significantly lower disease-specific quality of life [34]. Therefore, nowadays, personalised psychotherapy and counselling are suggested for FD management alongside conventional treatments [3, 33]. In addition, FD was found to be closely related to irritable bowel syndrome (IBS), possibly due to complex gastrointestinal immune responses after *H.* *pylori* infections or alterations in gastrointestinal microbiota [35]. Considering the above, the senior TCM practitioners might want to identify FD patients with a higher chance of having those common comorbidities or a poorer quality of life in order to offer them additional TCM interventions, psychiatric assessments, and specialist referrals in a timely manner.

TCM diagnostic patterns could be useful in facilitating case-finding of patients with potential comorbidities in routine practice. For instance, a study illustrated that idiopathic tinnitus patients with liver fire flaming upward or phlegm-fire stagnation tended to have moderate to severe anxiety symptoms [36]. Those with spleen and stomach qi deficiency or insufficiency of kidney essence were more likely to experience moderate to severe depressive symptoms [36]. Although a fundamental theory in TCM states the importance of factoring in individual differences like comorbidities when tailoring treatments [13, 14], the TCM expert consensus for FD management in China [10] did not mention the relationships between specific TCM diagnostic patterns and common comorbidities. The practitioners could perform regression analyses to investigate the associations between diagnostic patterns and FD comorbidities, including depression, anxiety, and IBS. Such knowledge will inform case-finding decisions in TCM practice, of which additional assessments would be offered to patients with specific TCM diagnostic pattern(s). Additional treatments or appropriate referrals could then be arranged to enhance care quality.

#### Clinical problem 4: generalisability of traditional Chinese medicine diagnostic pattern differentiation rules

Let us assume that score-based pattern differentiation rules were developed for Hong Kong patients using LTA. Some TCM practitioners in the team were invited to serve as visiting clinicians in Hunan, China, to share their experiences, but they were worried about the generalisability of the rules beyond patients for whom the rules were originally developed. It reflects another fundamental theory in TCM that diagnostic patterns are expected to vary geographically [13, 14]. If this theory were valid, the pattern differentiation rules of FD developed for patients in Hong Kong might not apply to patients in other geographical locations, like Hunan. This highlights the need to establish location-specific pattern differentiation rules using data from cross-sectional studies on different populations.

This TCM theory has been supported by current empirical research. A study showed that 51.4% of the German participants with menopausal symptoms had a TCM diagnostic pattern of kidney yang deficiency, which sharply contrasted with a prevalence of 5.7% among Chinese menopausal women [37]. On the other hand, 74.3% of the Chinese participants had kidney yin deficiency compared to 17.1% of the German participants [37]. The existence and clinical relevance of geographical variations can be examined by developing separate pattern differentiation rules for FD patients in Hong Kong and Hunan. It will allow comparisons across the two geographical locations on (i) the distributions of different TCM diagnostic patterns and (ii) the variations in clinical features that constitute the same TCM diagnostic patterns. Results on the distributions can inform the pre-test probabilities of TCM diagnostic patterns of FD patients in Hong Kong and Hunan, allowing location-specific treatment choices. Also, results on the variations in clinical features that constitute the same TCM diagnostic patterns enable the understanding of how geographical locations influence the essentialness of different clinical features in pattern differentiation.

## Objectives

In this diagnostic cross-sectional study on FD patients, we aimed to response to the four clinical problems described above by (i) establishing the score-based differentiation rules using LTA, (ii) discovering the co-existence of TCM diagnostic patterns and their distributions, (iii) revealing the associations between TCM diagnostic patterns of FD and common comorbidities, and (iv) comparing the prevalence of different TCM diagnostic patterns in Hong Kong and Hunan, as well as geographical variations in constituting clinical features on the same patterns. The definitions of key terms used in this study are listed in Additional file 1: Appendix 1.

## Methods

### Settings

Following the recommendations from the Quality Assessment of Diagnostic Accuracy Studies 2 [38], FD patients presenting in the gastrointestinal outpatient departments in Hong Kong (Prince of Wales Hospital, *n* = 250) and Hunan (Xiangya Hospital, *n* = 150) were sampled consecutively. Newspaper advertisements were also used for subject recruitment in Hong Kong to improve generalisability beyond the designated hospital. In both locations, our recruitment promotions only focused on the local communities. Potential subjects in Hong Kong and Hunan must be domiciled in respective locations. In brief consultations or phone-screening sessions, potential subjects were screened for eligibility by trained TCM practitioners. Medical records were then accessed and reviewed for eligibility confirmation. The recruitment period was from December 2020 to May 2021. This study was approved by the Joint Chinese University of Hong Kong—New Territories East Cluster Clinical Research Ethics Committee (Reference number: CREC 2018.325).

### Participants

Subjects who fulfilled all the following criteria were included:

1. Completed oesophagogastroduodenoscopy within ten years with *H.* *pylori*-negative results, or had tested positive for *H.* *pylori* and had completed the course for *H.* *pylori* eradication;
2. Having symptoms that fulfilled the Ford et al. Reference Standard for FD [39], with no restriction on the subtypes of PDS, EPS, or overlapping of PDS and EPS;
3.  ≥ 18 years of age; AND
4. Provided written informed consent.

Subjects who fulfilled any of the following criteria were excluded:

1. Had a diagnosis of organic oesophageal or gastric diseases in the past month, including oesophagitis, gastro-oesophageal reflux disease, peptic ulcer, and predominant heartburn or acid regurgitation;
2. Presence of unremoved stomach polyps;
3. Ever received major abdominal surgery (i.e., appendectomy, gastrectomy, removal of gastric lymph nodes, cholecystectomy, and removal of abdominal cancers);
4. Pregnant at the time of enrolment;
5. Experiencing major physical illnesses (i.e., malignancy and serious infections); OR
6. Refused to provide written informed consent.

### Data sources and measurements

At both sites, participants were invited to complete an online questionnaire of four sections. The first section collected data on basic demographic and clinical characteristics. The second section evaluated their FD subtype via the Ford et al. Reference Standard for FD [39]. IBS status of the participants was also assessed by the Rome IV Diagnostic Questionnaire for Adult Functional Gastrointestinal Disorders [1]. The third section contained a fifty-five-item Traditional Chinese Medicine Clinical Feature Questionnaire for Functional Dyspepsia (TCMQ-FD), designed to collect participants’ self-reported clinical feature data. Participants were invited to rate the severity of each clinical feature on a five-point Likert scale, with a higher numerical rating indicating higher severity. Targeted for TCM diagnostic pattern differentiation, the items were developed from two sources: (i) a systematic review of TCM diagnostic instruments for FD [22] and (ii) the 2017 Chinese Medicine Expert Consensus on Functional Dyspepsia Diagnosis in China [10]. The final section assessed psychiatric comorbidities and disease-specific quality of life of FD. Participants’ depressive and anxiety symptoms were measured by the Patient Health Questionnaire-9 (PHQ9) [40] and the General Anxiety Disorder-7 (GAD7) [41], respectively, in validated Chinese versions. Disease-specific quality of life was evaluated using the validated Chinese version of the Nepean Dyspepsia Index (NDI) [42].

### Statistical methods

#### Latent tree analysis

We used three sets of TCMQ-FD clinical feature data corresponding to three study samples to derive score-based pattern differentiation rules for TCM diagnostic patterns of FD. The dataset derived from the overall sample refers to the data collected from all 400 participants. The dataset for the Hong Kong sample is defined as the data collected from the 250 participants in Hong Kong. The dataset for the Hunan sample refers to the data collected from the 150 participants in Hunan. On the *Lantern* software [43, 44], we analysed the overall sample dataset using LTA [26], a quantitative approach consisting of five steps as summarised in Table 1:

**Table 1:** Preparation and execution of latent tree analysis

Step^a^		Procedure
(i)	Statistical pattern discovery	Build three independent global latent tree models on the Lantern software Choose the model with the best BIC score for subsequent steps Obtain probabilistic co-occurring clinical features from each latent variable
(ii)	Statistical pattern interpretation	Examine the quantitative relationships between latent variables and constituting clinical features by checking relevant probability distributions on Lantern Determine the TCM diagnostic pattern connotations for the latent variables from clinical perspective with TCM expertise Generate a list of potential TCM diagnostic patterns
(iii)	Traditional Chinese Medicine diagnostic pattern identification	Based on TCM expertise, select only the potential TCM diagnostic patterns that contain all essential clinical features for subsequent steps Discard those that do not contain all essential clinical features
(iv)	Traditional Chinese Medicine diagnostic pattern quantification	Construct a local latent tree model for each selected TCM diagnostic pattern on Lantern
(v)	Traditional Chinese Medicine pattern differentiation rule derivation	Apply the local latent tree models to classify the participants Assign a soft label to each participant based on the probability of belonging to each TCM diagnostic pattern Derive score-based differentiation rules using the Naïve Bayes approach^b^

*BIC* Bayesian information criterion, *TCM* Traditional Chinese Medicine, *TCMQ-FD* Traditional Chinese Medicine Clinical Feature Questionnaire for Functional Dyspepsia

^a^This study involved three datasets. Steps (i) to (iv) were performed using the overall sample dataset. In Step (v), the local latent tree models constructed were used to classify and label the participants in the Hong Kong sample and the Hunan sample, and derive relevant pattern differentiation rules for the two samples

^b^See Zhang et al. [26] for details

(i) Statistical pattern discovery: The data were dichotomised based on a cut-off score of four. In other words, if a patient rated four or above on a clinical feature, that patient was deemed to express that clinical feature in a significant manner. The dichotomised data were fed to Lantern. The output was a global latent tree model (LTM), a tree-structured probabilistic graphical model with latent variables at the internal nodes and the observed variables (i.e., patient-reported clinical features) located at the leaf nodes [25–27]. The latent variables captured the probabilistic co-occurrence patterns among the clinical features. Three independent global LTMs were constructed for model fit comparisons, with the one having the best Bayes information criterion score selected for the next step.

(ii) Statistical pattern interpretation: Three TCM practitioners (LH, FFH, and VCHC) were invited to determine the TCM diagnostic pattern connotations of the latent variables via discussions. Along with their TCM domain knowledge [13, 14], the practitioners were allowed to make reference to the systematic review of the TCM diagnostic instruments for FD [22] and the TCM expert consensus for FD management in China [10] for the task. A list of potential TCM diagnostic patterns was created as a result.

(iii) TCM diagnostic pattern identification: The three TCM practitioners were then asked to determine, for each potential TCM diagnostic pattern from the previous step, whether all its essential clinical features were present in the data. A TCM diagnostic pattern was kept only if all TCM practitioners answered positively.

(iv) TCM diagnostic pattern quantification: For each remaining TCM diagnostic pattern, a local LTM (also known as a “joint-clustering model”) was built on Lantern using relevant clinical features and latent variables. A local LTM was used to probabilistically partition the overall sample into two clusters and gave a definition of the TCM diagnostic pattern over the overall sample dataset [26].

(v) TCM pattern differentiation rule derivation: The local LTMs were used to classify the participants in the overall sample. Each participant was assigned a soft label that specified the probability of belonging to a particular TCM diagnostic pattern. The soft labels were subsequently adopted to derive pattern differentiation rules using the Naïve Bayes approach, as illustrated in Zhang et al. [26]. According to the original procedure for LTA [26], pattern differentiation rules are supposed to be derived from local LTMs on Lantern directly instead of from soft labels. As this study involved three samples, namely the overall sample, the Hong Kong sample, and the Hunan sample, following the original procedure would mean going through the five steps three times, once for each sample. This would result in three different definitions for each TCM diagnostic pattern and, more importantly, would suffer from insufficient data. Therefore, we carried out LTA only on the overall sample dataset and derived pattern differentiation rules for the three samples from the soft labels.

#### Prevalence of traditional Chinese medicine diagnostic patterns of functional dyspepsia across the three samples

We differentiated the TCM diagnostic patterns from the overall sample (*n* = 400), the Hong Kong sample (*n* = 250), and the Hunan sample (*n* = 150) using corresponding pattern differentiation rules. We summarised and compared the number of TCM diagnostic pattern(s) differentiated in individual participants and the prevalence of different TCM diagnostic patterns of FD across the three samples.

#### Co-existence of traditional Chinese medicine diagnostic patterns of functional dyspepsia

Using the overall sample dataset, we performed multivariate logistic regressions to examine the relationships between each pair of TCM diagnostic patterns. The regression model for each pair of TCM diagnostic patterns of concern was adjusted for the remaining TCM diagnostic patterns. Results were presented in adjusted odds ratios (AORs) and 95% confidence intervals (CIs). Hosmer and Lemeshow tests were conducted to evaluate the goodness of fit of the models. A *p* value of larger than 0.10 indicated an adequate fit. We also presented the counts and prevalence of the co-existing pairs of TCM diagnostic patterns differentiated in the overall sample.

#### Associations between traditional Chinese medicine diagnostic patterns of functional dyspepsia and its common comorbidities and disease-specific quality of life

Multivariate logistic regressions were conducted to quantify the associations between different TCM diagnostic patterns and FD subtypes, comorbid IBS, geographic locations, and PHQ9, GAD7, and NDI scores. Pearson and Deviance tests were performed to assess the models’ goodness of fit. A *p* value of larger than 0.10 denoted an adequate fit.

## Results

### Participants

**Fig. 1:**
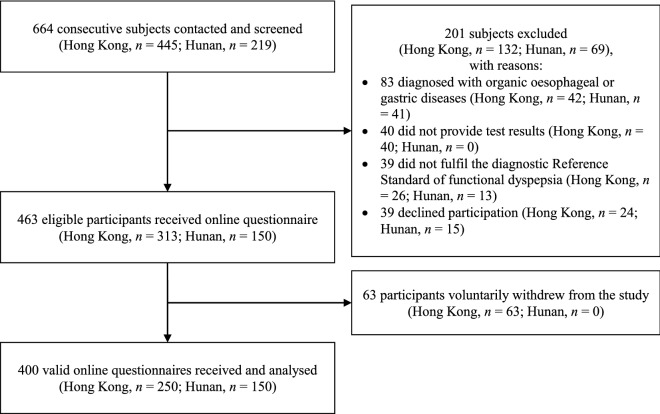
Flow of study participants

Figure 1 presents the flow of the participants of this diagnostic cross-sectional study. We screened a total of 445 subjects in Hong Kong. Among them, 132 were excluded due to organic oesophageal or gastric disease diagnoses (*n* = 42), unavailable test results (*n* = 40), failure in fulfilling the diagnostic Reference Standard (*n* = 26), or declined participation (*n* = 24). Then, we provided 313 eligible participants with access to the online questionnaire for this study. In the end, a total of 250 valid online questionnaires were received and analysed, following the voluntary withdrawal of sixty-three participants.

On the other hand, we screened a total of 219 subjects in Hunan. Among them, sixty-nine were excluded due to organic oesophageal or gastric disease diagnoses (*n* = 41), declined participation (*n* = 15), or unavailability of test results (*n* = 13). We provided 150 eligible participants with access to the online questionnaire. Ultimately, we were able to receive and analyse all 150 online questionnaires.

In summary, the response rates in Hong Kong and Hunan were 79.9% and 100%, respectively. The overall response rate was 86.4%. No missing data were identified in both locations. Table 2 shows the demographic and clinical characteristics of the 400 participants.

**Table 2:** Basic demographical and clinical characteristics of participants

	Hong Kong sample (*n* = 250)	Hunan sample (*n* = 150)
Age (years), mean (SD)	51.4 (13.0)	45.2 (13.7)
Female, *n* (%)	199 (79.6)	101 (67.3)
Body mass index (kg/m^2^), mean (SD)	20.8 (7.7)	22.1 (4.6)
Duration of symptoms (years), mean (SD)	3.6 (5.3)	2.3 (3.4)
Symptom subtype of functional dyspepsia		
Postprandial distress syndrome only	70 (28.0)	28 (18.7)
Epigastric pain syndrome only	18 (7.2)	28 (18.7)
Overlapping between two subtypes	162 (64.8)	94 (62.6)
Self-reported duration of symptoms, *n* (%)		
≥ 5 years	57 (22.8)	18 (12.0)
< 5 years	193 (77.2)	132 (88.0)
PHQ9 total score^a^		
Mean (SD)	7.0 (4.9)	7.1 (6.4)
Depression (cut-off 10), *n* (%)	67 (26.8)	51 (34.0)
GAD7 total score^b^		
Mean (SD)	5.8 (5.3)	6.6 (5.7)
Anxiety (cut-off 10), *n* (%)	47 (18.8)	41 (27.3)
NDI results, mean (SD)		
Symptom severity^c^	44.8 (27.7)	53.6 (32.6)
Eating/drinking^d^	64.0 (23.2)	68.9 (24.4)
Sleep^d^	62.9 (29.6)	67.8 (30.0)
Knowledge/control^d^	70.2 (21.0)	67.7 (24.7)
Interference^d^	69.3 (20.0)	70.2 (21.7)
Total QoL score^d^	66.6 (20.0)	68.6 (21.6)
With concomitant IBS diagnosis^e^, *n* (%)	45 (18.0)	26 (17.3)

*GAD7* 7-item generalised anxiety disorder scale, *IBS* Irritable bowel syndrome, *NDI* Nepean Dyspepsia Index, *PHQ9* 9-item patient’s health questionnaire, *QoL* Quality of life, *SD* Standard deviation

^a^Maximum score = 27 with lower score indicating lower severity

^b^Maximum score = 21 with lower score indicating lower severity

^c^Maximum score = 195 with lower score indicating less symptom

^d^Maximum score = 100 with higher score representing better quality of life

^e^IBS was positive in this study when the following Rome IV criteria were fulfilled in the past three months at enrolment: (i) recurrent abdominal pain at least weekly; (ii) pain is associated with two or more of the following criteria: (a) at least 30% of occasions related to defecation; (b) at least 30% of occasions associated with a change in frequency of stool; or (c) at least 30% of occasions associated with a change in form (appearance) of stool; and (iii) symptom onset at least six months prior to diagnosis

### Latent tree models for the traditional Chinese medicine diagnostic patterns of functional dyspepsia

**Fig. 2:**
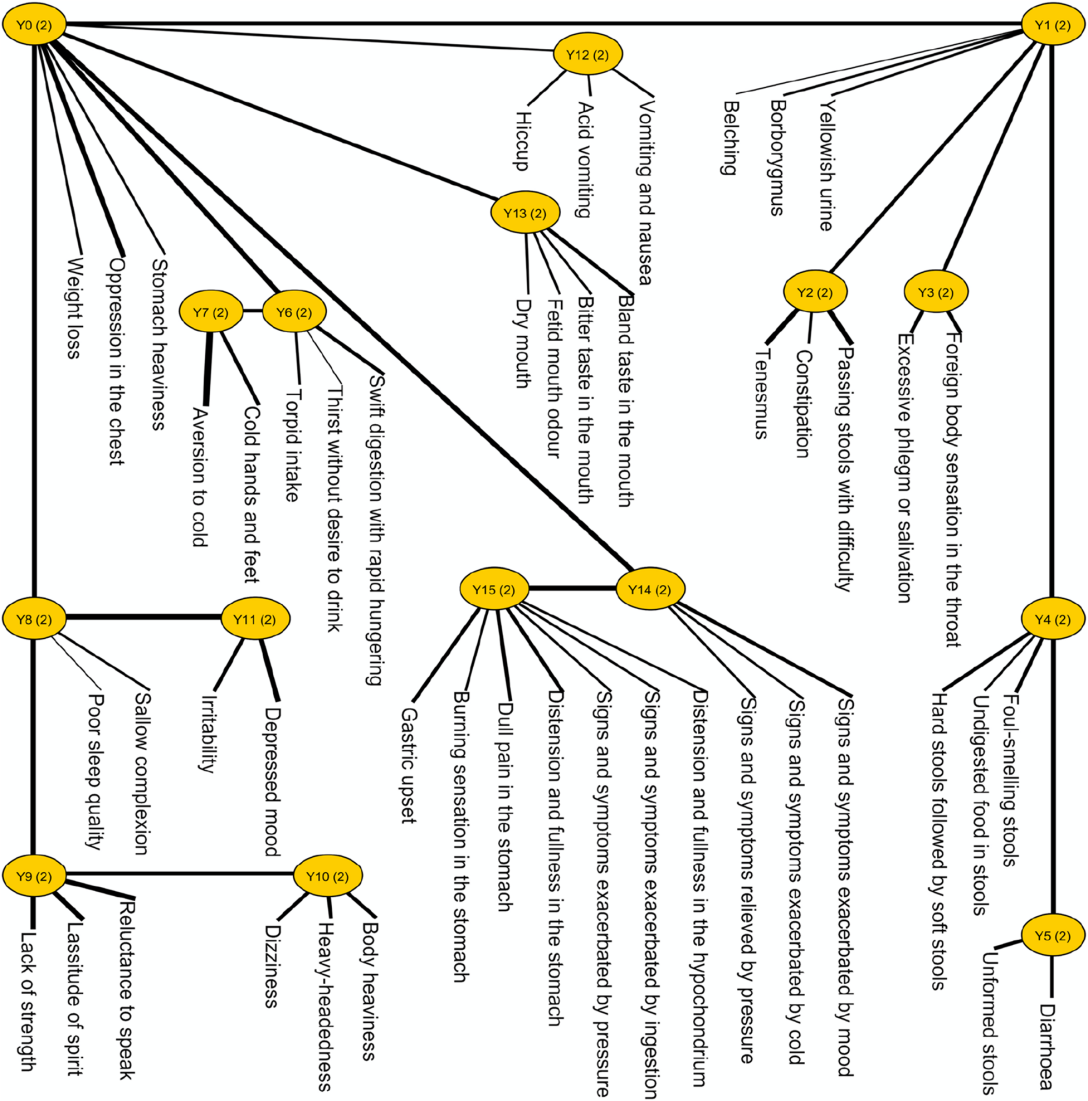
Global latent tree model for Traditional Chinese Medicine clinical features of functional dyspepsia constructed using the overall sample dataset (*n* = 400). Latent tree model is an undirected tree with the observed variables located at the leaf nodes and the latent variables at the internal nodes. It explains the relationships between the observed variables (i.e., clinical features) and their latent variables using conditional probability distributions. “Y”s are the latent variables in the latent tree model. The number in parentheses is the number of clusters in the latent variables from the probabilistic partition of participants. All latent variables in the above model contained two participant clusters. Based on the manifestation of probabilistic co-occurring clinical features, one of the clusters included participants that were classified into that latent variable, while the other included those that were not classified into that latent variable

A global LTM was constructed for the TCM clinical features of FD using the overall sample dataset (Fig. 2). The *Y*-variables in the model are the latent variables of their observed variables (i.e., clinical features) [26]. The number in parentheses next to each latent variable indicates the number of possible clusters from the probabilistic partition of participants [26]. In other words, all latent variables in the global LTM had two clusters of participants (Fig. 2). Based on the manifestation of probabilistic co-occurring clinical features, one of the clusters included participants that were classified into that latent aspect, while the other included those that were not classified into that latent aspect. The probabilistic co-occurring clinical features in the latent variables of the global LTM are listed in Additional file 1: Appendix 2.

**Fig. 3:**
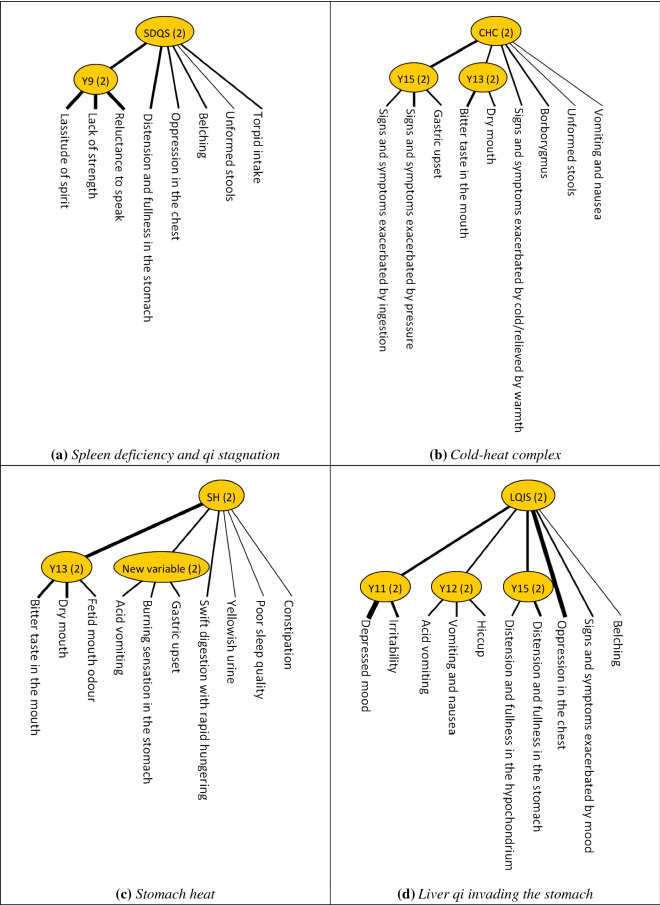

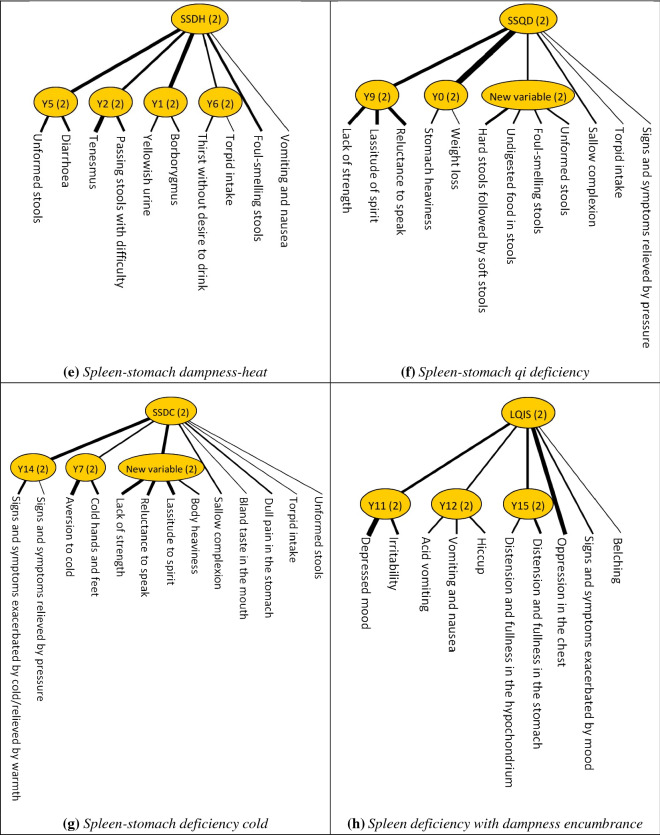
Latent tree models for Traditional Chinese Medicine diagnostic patterns of functional dyspepsia constructed using the overall sample dataset (*n* = 400). “Y”s and “New variable”s are the latent variables in the latent tree models. The number in parentheses is the number of clusters in the latent variables from the probabilistic partition of participants. All latent variables in the above model contained two participant clusters. Based on the manifestation of probabilistic co-occurring clinical features, one of the clusters included participants that were classified into that latent variable, while the other included those that were not classified into that latent variable

A total of eight LTMs were finalised from the LTA, corresponding to eight TCM diagnostic patterns of FD (Fig. 3): (a) spleen deficiency and qi stagnation; (b) cold-heat complex; (c) stomach heat; (d) liver qi invading the stomach; (e) spleen-stomach dampness-heat; (f) spleen-stomach qi deficiency; (g) spleen-stomach deficiency cold; and (h) spleen deficiency with dampness encumbrance.

## Main results

### Score-based pattern differentiation rules for traditional Chinese medicine diagnostic patterns of functional dyspepsia

A score-based pattern differentiation rule was established for each TCM diagnostic pattern using the overall sample dataset (Table 3). The differentiation of seven out of eight TCM diagnostic patterns of FD relied on the co-existence of at least two constituting clinical features. For instance, if a patient was to be diagnosed with cold-heat complex, that patient must report at least borborygmus (numerical score: 6.5) and vomiting and nausea (numerical score: 3.5) so as to meet the differentiation threshold of 10.0 (Table 3). However, if a patient reported having depressed mood, that patient could already be diagnosed with liver qi invading the stomach, given that the numerical score of the clinical feature was as high as 10.7 while the threshold of the TCM diagnostic pattern was only 10.0 (Table 3).

**Table 3:** Comparisons of score-based differentiation rules of Traditional Chinese Medicine diagnostic patterns of functional dyspepsia across the three samples

Overall sample (*n* = 400)		Hong Kong sample (*n* = 250)		Hunan sample (*n* = 150)	
Clinical feature	Score	Clinical feature	Score	Clinical feature	Score
**Spleen deficiency and qi stagnation**					
Distension and fullness in the stomach	5.5	Distension and fullness in the stomach	7.6	Distension and fullness in the stomach	3.5
Oppression in the chest	4.2	Oppression in the chest	5.3	Lack of strength	2.9
Lack of strength	3.6	Lack of strength	4.1	Reluctance to speak	2.8
Reluctance to speak	3.6	Reluctance to speak	3.9	Oppression in the chest	2.6
Torpid intake	2.2	Torpid intake	1.8	Torpid intake	2.6
Lassitude of spirit	1.9	Belching	1.5	Lassitude of spirit	2.5
Belching	1.5	Lassitude of spirit	1.4	Belching	1.4
Unformed stools	0.9	Unformed stools	0.8	Unformed stools	0.8
**Cold-heat complex**					
Signs and symptoms exacerbated by pressure	6.9	Signs and symptoms exacerbated by pressure	7.6	Borborygmus	8.3
Borborygmus	6.5	Borborygmus	6.0	Signs and symptoms exacerbated by pressure	6.0
Bitter taste in the mouth	4.7	Bitter taste in the mouth	5.5	Vomiting and nausea	4.0
Signs and symptoms exacerbated by ingestion	4.3	Signs and symptoms exacerbated by ingestion	5.5	Bitter taste in the mouth	4.0
Gastric upset	4.0	Gastric upset	4.4	Unformed stools	3.2
Vomiting and nausea	3.5	Vomiting and nausea	3.1	Signs and symptoms exacerbated by ingestion	3.1
Unformed stools	3.0	Unformed stools	2.9	Gastric upset	2.5
Signs and symptoms exacerbated by cold	2.5	Signs and symptoms exacerbated by cold	2.7	Dry mouth	2.4
Dry mouth	2.2	Dry mouth	1.9	Signs and symptoms exacerbated by cold	2.2
**Stomach heat**					
Burning sensation in the stomach	9.6	Bitter taste in the mouth	8.5	Burning sensation in the stomach	7.7
Acid vomiting	8.5	Burning sensation in the stomach	7.7	Acid vomiting	6.9
Bitter taste in the mouth	7.7	Acid vomiting	6.2	Bitter taste in the mouth	6.6
Gastric upset	4.1	Fetid mouth odour	4.9	Gastric upset	5.6
Dry mouth	3.8	Gastric upset	3.7	Dry mouth	4.2
Fetid mouth odour	3.4	Dry mouth	3.6	Swift digestion with rapid hungering	2.7
Swift digestion with rapid hungering	3.2	Swift digestion with rapid hungering	3.6	Constipation	2.6
Yellowish urine	2.6	Yellowish urine	3.0	Fetid mouth odour	2.0
Constipation	2.4	Constipation	2.2	Poor sleep quality	2.0
Poor sleep quality	1.8	Poor sleep quality	1.7	Yellowish urine	1.8
**Liver qi invading the stomach**					
Depressed mood	10.7	Depressed mood	11.9	Depressed mood	7.0
Oppression in the chest	7.6	Oppression in the chest	8.3	Oppression in the chest	5.7
Acid vomiting	5.8	Acid vomiting	7.8	Acid vomiting	4.3
Irritability	4.7	Irritability	4.9	Irritability	4.2
Vomiting and nausea	3.5	Distension and fullness in the hypochondrium	3.3	Vomiting and nausea	4.0
Distension and fullness in the hypochondrium	3.4	Vomiting and nausea	2.9	Signs and symptoms exacerbated by mood	3.6
Signs and symptoms exacerbated by mood	2.9	Signs and symptoms exacerbated by mood	2.7	Distension and fullness in the hypochondrium	3.0
Hiccup	2.1	Distension and fullness in the stomach	0.7	Belching	2.5
Distension and fullness in the stomach	1.1	Hiccup	0.6	Hiccup	2.2
Belching	0.5	Belching	− 0.3	Distension and fullness in the stomach	1.7
**Spleen-stomach dampness-heat**					
Tenesmus	7.6	Tenesmus	8.1	Tenesmus	6.9
Passing stools with difficulty	5.0	Passing stools with difficulty	6.1	Unformed stools	4.0
Unformed stools	4.8	Unformed stools	5.3	Diarrhoea	3.8
Foul-smelling stools	3.9	Foul-smelling stools	4.4	Passing stools with difficulty	3.4
Diarrhoea	3.4	Diarrhoea	3.3	Foul-smelling stools	3.1
Yellowish urine	2.0	Borborygmus	2.2	Yellowish urine	1.8
Borborygmus	1.9	Yellowish urine	2.1	Vomiting and nausea	1.4
Vomiting and nausea	1.4	Thirst without desire to drink	1.6	Borborygmus	1.1
Thirst without desire to drink	1.0	Vomiting and nausea	1.4	Torpid intake	− 0.4
Torpid intake	− 0.1	Torpid intake	0.0	Thirst without desire to drink	0.0
**Spleen-stomach qi deficiency**					
Reluctance to speak	8.6	Lack of strength	9.9	Lack of strength	6.2
Lack of strength	8.1	Reluctance to speak	8.8	Reluctance to speak	5.5
Stomach heaviness	5.5	Sallow complexion	6.5	Stomach heaviness	4.6
Sallow complexion	5.4	Stomach heaviness	5.4	Sallow complexion	4.1
Undigested food in stools	3.8	Undigested food in stools	4.6	Lassitude of spirit	4.0
Hard stools followed by soft stools	3.8	Hard stools followed by soft stools	4.5	Weight loss	3.1
Weight loss	3.0	Foul-smelling stools	2.7	Hard stools followed by soft stools	2.9
Lassitude of spirit	2.9	Lassitude of spirit	2.6	Undigested food in stools	2.8
Foul-smelling stools	2.3	Weight loss	2.3	Torpid intake	2.6
Unformed stools	1.6	Signs and symptoms relieved by pressure	1.9	Foul-smelling stools	1.8
Signs and symptoms relieved by pressure	1.6	Unformed stools	1.8	Unformed stools	1.4
Torpid intake	1.3	Torpid intake	0.4	Signs and symptoms relieved by pressure	1.2
**Spleen-stomach deficiency cold**					
Reluctance to speak	8.1	Lack of strength	8.0	Reluctance to speak	5.3
Lack of strength	6.5	Reluctance to speak	7.3	Lack of strength	5.0
Sallow complexion	3.9	Sallow complexion	4.6	Body heaviness	3.5
Body heaviness	3.4	Bland taste in the mouth	3.6	Lassitude of spirit	3.4
Bland taste in the mouth	2.9	Body heaviness	3.3	Sallow complexion	3.1
Lassitude of spirit	2.7	Lassitude of spirit	2.4	Torpid intake	2.5
Dull pain in the stomach	2.1	Cold hands and feet	2.2	Bland taste in the mouth	2.2
Aversion to cold	2.1	Dull pain in the stomach	2.1	Aversion to cold	2.1
Cold hands and feet	1.8	Aversion to cold	2.1	Dull pain in the stomach	1.9
Signs and symptoms exacerbated by cold	1.7	Signs and symptoms exacerbated by cold	2.0	Signs and symptoms exacerbated by cold	1.5
Signs and symptoms relieved by pressure	1.5	Signs and symptoms relieved by pressure	1.8	Cold hands and feet	1.4
Torpid intake	1.2	Unformed stools	0.7	Signs and symptoms relieved by pressure	1.2
Unformed stools	0.8	Torpid intake	0.5	Unformed stools	0.9
**Spleen deficiency with dampness encumbrance**					
Unformed stools	5.2	Unformed stools	5.4	Tenesmus	4.7
Tenesmus	4.8	Bland taste in the mouth	5.4	Unformed stools	4.6
Bland taste in the mouth	4.4	Tenesmus	4.9	Bland taste in the mouth	3.5
Excessive phlegm or salivation	4.4	Excessive phlegm or salivation	4.8	Foul-smelling stools	3.5
Foul-smelling stools	3.8	Passing stools with difficulty	4.3	Excessive phlegm or salivation	3.5
Vomiting and nausea	3.7	Foul-smelling stools	3.9	Vomiting and nausea	3.3
Passing stools with difficulty	3.6	Vomiting and nausea	3.8	Diarrhoea	3.0
Heavy-headedness	3.2	Heavy-headedness	3.4	Heavy-headedness	2.7
Dizziness	2.7	Dizziness	3.4	Passing stools with difficulty	2.5
Diarrhoea	2.7	Foreign body sensation in the throat	2.6	Borborygmus	2.3
Borborygmus	2.5	Borborygmus	2.5	Foreign body sensation in the throat	2.2
Foreign body sensation in the throat	2.5	Body heaviness	2.5	Body heaviness	2.2
Body heaviness	2.4	Diarrhoea	2.4	Dizziness	2.0
Thirst without desire to drink	1.6	Thirst without desire to drink	1.5	Thirst without desire to drink	1.6
Torpid intake	0.8	Torpid intake	0.5	Torpid intake	1.2

For ease of comparison, the differentiation thresholds were standardised to 10.0, and the numerical score for each clinical feature was scaled accordingly

### Comparing the pattern differentiation rules across the three samples

Table 3 also compares the pattern differentiation rules and the constituting clinical features of the eight TCM diagnostic patterns of FD across the overall, Hong Kong, and Hunan samples. For ease of comparison, the differentiation thresholds were standardised to 10.0, and the numerical score for each clinical feature was scaled accordingly. It is worth noting that none of the TCM diagnostic patterns shared the same prioritisation of constituting clinical features across all three samples. This illustrates that the importance of each constituting clinical feature for differentiating the same TCM diagnostic patterns varies across different populations. For example, in terms of numerical score, distension and fullness in the stomach, oppression in the chest, and lack of strength were the top three clinical features for differentiating spleen deficiency and qi stagnation in the overall and Hong Kong samples. Hence, patients with these clinical features would already be diagnosed with that TCM diagnostic pattern. In the Hunan sample, distension and fullness in the stomach, lack of strength, and reluctance to speak were the top three clinical features for the same diagnostic pattern. Under the Hunan pattern differentiation rule, patients who reported having only distension and fullness in the stomach, oppression in the chest, and lack of strength would not be diagnosed with spleen deficiency and qi stagnation.

### Prevalence of different diagnostic patterns across the three samples

The pattern differentiation rules were able to differentiate at least one TCM diagnostic pattern in 70.7%, 73.6%, and 64.0% of the participants in the overall (*n* = 400), Hong Kong (*n* = 250), and Hunan (*n* = 150) samples, respectively (Table 4). Only one TCM diagnostic pattern was differentiated in 14.5%, 14.0%, and 11.3% of the participants in the three samples, respectively (Table 4). It is also worth mentioning that 5.3%, 5.6%, and 6.0% of the participants in the three samples were diagnosed to express eight co-existing TCM diagnostic patterns (Table 4).

**Table 4:** Prevalence of traditional Chinese medicine diagnostic patterns of functional dyspepsia across the three samples

	Overall sample *n* (%)	Hong Kong sample *n* (%)	Hunan sample *n* (%)
**Number of TCM diagnostic pattern(s) on individual participants**			
No TCM diagnostic pattern	117 (29.3)	66 (26.4)	54 (36.0)
1 TCM diagnostic pattern	58 (14.5)	35 (14.0)	17 (11.3)
2 TCM diagnostic patterns	42 (10.5)	32 (12.8)	15 (10.0)
3 TCM diagnostic patterns	38 (9.5)	27 (10.8)	6 (4.0)
4 TCM diagnostic patterns	27 (6.8)	17 (6.8)	11 (7.3)
5 TCM diagnostic patterns	33 (8.3)	20 (8.0)	18 (12.0)
6 TCM diagnostic patterns	33 (8.3)	20 (8.0)	9 (6.0)
7 TCM diagnostic patterns	31 (7.8)	19 (7.6)	11 (7.3)
8 TCM diagnostic patterns	21 (5.3)	14 (5.6)	9 (6.0)
**Distribution of TCM diagnostic pattern among participants with at least one diagnosis**			
Spleen deficiency with dampness encumbrance	159 (56.2)	106 (57.6)	42 (43.8)
Liver qi invading the stomach	158 (55.8)	82 (44.6)	74 (77.1)
Spleen-stomach qi deficiency	153 (54.1)	87 (47.3)	63 (65.6)
Cold-heat complex	148 (52.3)	110 (59.8)	42 (43.8)
Stomach heat	147 (51.9)	88 (47.8)	58 (60.4)
Spleen-stomach deficiency cold	145 (51.2)	78 (42.4)	63 (65.6)
Spleen deficiency and qi stagnation	134 (47.3)	105 (57.1)	42 (43.8)
Spleen-stomach dampness-heat	68 (24.0)	57 (31.0)	18 (18.8)

*TCM* traditional Chinese medicine

Prevalence of Traditional Chinese Medicine diagnostic patterns in a sample was calculated based on the standardised score-based differentiation rules of that particular sample as presented in Table 3

Table 4 also shows the distribution of different TCM diagnostic patterns among participants with at least one TCM diagnosis across the three samples. Spleen deficiency with dampness encumbrance was the most common TCM diagnostic pattern among participants with at least one diagnosis in the overall sample, with a prevalence of 56.2%. Cold-heat complex and liver qi invading the stomach were the most common TCM diagnostic patterns in the Hong Kong (59.8%) and Hunan (77.1%) samples, respectively. Spleen-stomach dampness-heat was the least prevalent diagnostic pattern across the three samples.

### Co-existence of traditional Chinese medicine diagnostic patterns

Results of the logistic regressions assessing the relationships between TCM diagnostic patterns in the overall sample are presented in Table 5. The *p* values of the Hosmer and Lemeshow tests were larger than 0.10, indicating good model fit. Fourteen pairs of TCM diagnostic patterns were observed with significant positive associations, with exceptionally high AORs (> 5.00) revealed in three pairs. Spleen-stomach deficiency cold tended to co-exist with spleen-stomach qi deficiency (AOR: 53.23; 95% CI: 21.77–130.16) and vice versa (AOR: 49.61; 95% CI: 20.96–117.44). Spleen deficiency and qi stagnation tended to co-exist with spleen-stomach deficiency cold (AOR: 8.73; 95% CI: 3.52–21.68) and vice versa (AOR: 8.66; 95% CI: 3.52–21.30). Spleen deficiency and qi stagnation tended to co-exist with cold-heat complex (AOR: 6.07; 95% CI: 2.86–12.90) and vice versa (AOR: 6.03; 95% CI: 2.84–12.80).

**Table 5:** Logistic regressions on the associations between Traditional Chinese Medicine diagnostic patterns using the overall sample dataset (*n* = 400)

Independent variable	Dependent variable							
	SDDE^*^	LQIS^*^	SSQD^*^	CHC^*^	SH^*^	SSDC^*^	SDQS^*^	SSDH^*^
SDDE		**2.04 (1.07–3.87)**	**2.86 (1.14–7.16)**	**2.23 (1.21–4.10)**	**1.98 (1.08–3.63)**	1.21 (0.45–3.27)	1.18 (0.53–2.64)	–^a^
LQIS	**2.11 (1.10–4.03)**		0.90 (0.38–2.15)	0.76 (0.41–1.43)	**3.27 (1.85–5.78)**	**3.39 (1.45–7.95)**	**3.87 (1.90–7.87)**	1.31 (0.59–2.95)
SSQD	**2.79 (1.03–7.52)**	0.94 (0.40–2.20)		1.16 (0.48–2.82)	**2.51 (1.09–5.77)**	**49.61 (20.96–117.44)**	**3.61 (1.48–8.82)**	1.80 (0.60–5.42)
CHC	**2.34 (1.26–4.35)**	0.76 (0.40–1.41)	1.10 (0.45–2.70)		**3.43 (2.00–5.89)**	0.53 (0.20–1.43)	**6.03 (2.84–12.80)**	**2.28 (1.08–4.82)**
SH	**2.17 (1.18–3.98)**	**3.31 (1.87–5.87)**	**2.65 (1.11–6.28)**	**3.46 (2.01–5.93)**		0.70 (0.28–1.72)	1.17 (0.57–2.43)	1.19 (0.57–2.50)
SSDC	1.24 (0.43–3.51)	**3.46 (1.49–8.04)**	**53.23 (21.77–130.16)**	0.50 (0.19–1.33)	0.73 (0.30–1.78)		**8.66 (3.52–21.30)**	0.54 (0.17–1.70)
SDQS	1.06 (0.46–2.44)	**3.81 (1.89–7.69)**	**3.28 (1.32–8.16)**	**6.07 (2.86–12.90)**	1.09 (0.53–2.24)	**8.73 (3.52–21.68)**		0.80 (0.32–2.01)
SSDH	–^a^	1.38 (0.61–3.10)	1.97 (0.64–6.10)	**2.36 (1.11–4.99)**	1.10 (0.52–2.32)	0.58 (0.19–1.77)	0.65 (0.25–1.71)	

Logistic regression model for each pair of Traditional Chinese Medicine (TCM) diagnostic patterns was adjusted for the remaining TCM diagnostic patterns. Statistically significant results are bolded and underlined

*CHC* cold-heat complex, *LQIS* liver qi invading the stomach, *SDQS* spleen deficiency and qi stagnation, *SDDE* spleen deficiency with dampness encumbrance; SH, Stomach heat; SSDC, Spleen-stomach deficiency cold; SSDH, Spleen-stomach dampness-heat, *SSQD* spleen-stomach qi deficiency

^a^Results not shown due to statistical separation

^*^P values of the Hosmer and Lemeshow tests > 0.10, indicating good model fit

The prevalence of different pairs of co-existing patterns in the overall sample is illustrated in Table 6. The most common pair was spleen-stomach qi deficiency and spleen-stomach deficiency cold. They were found in 131 participants in the overall sample, with a prevalence of 32.8%. Although cold-heat complex and spleen-stomach dampness-heat constituted the least common co-existing patterns, they could still be diagnosed in forty-nine participants in the overall sample, with a prevalence of 12.3%.

**Table 6:** Prevalence of the co-existing Traditional Chinese Medicine diagnostic patterns in the overall sample (*n* = 400)

Co-existing Traditional Chinese Medicine diagnostic patterns of functional dyspepsia		Count	Prevalence (%)
Spleen-stomach qi deficiency	Spleen-stomach deficiency cold	131	32.8
Spleen-stomach qi deficiency	Spleen deficiency and qi stagnation	112	28.0
Spleen-stomach deficiency cold	Spleen deficiency and qi stagnation	111	27.8
Liver qi invading the stomach	Spleen-stomach deficiency cold	106	26.5
Spleen deficiency with dampness encumbrance	Spleen-stomach qi deficiency	104	26.0
Liver qi invading the stomach	Spleen deficiency and qi stagnation	102	25.5
Spleen deficiency with dampness encumbrance	Liver qi invading the stomach	101	25.3
Liver qi invading the stomach	Stomach heat	98	24.5
Spleen deficiency with dampness encumbrance	Cold-heat complex	97	24.3
Spleen deficiency with dampness encumbrance	Stomach heat	96	24.0
Spleen-stomach qi deficiency	Stomach heat	94	23.5
Cold-heat complex	Spleen deficiency and qi stagnation	89	22.3
Cold-heat complex	Stomach heat	84	21.0
Cold-heat complex	Spleen-stomach dampness-heat	49	12.3

### Associations between diagnostic patterns, location, disease subtype, comorbidities, and disease-specific quality of life

Multivariate logistic regression results on the associations between TCM diagnostic patterns with various health-related variables are reported in Table 7. The *p* values of the Pearson and Deviance tests are larger than 0.10, indicating good model fit. Participants located in Hong Kong were more likely to experience spleen deficiency and qi stagnation (AOR: 2.59; 95% CI: 1.05–6.40), spleen deficiency with dampness encumbrance (AOR: 2.34; 95% CI: 1.15–4.74), and cold-heat complex (AOR: 2.23; 95% CI: 1.18–4.21) than those located in Hunan. Compared to the participants with overlapping subtypes, those with the PDS subtype tended to have spleen-stomach qi deficiency (AOR: 3.20; 95% CI: 1.07–9.59).

**Table 7:** Logistic regressions between clinical variables and Traditional Chinese Medicine diagnostic patterns using the overall sample dataset (*n* = 400)

Clinical variable	TCM diagnostic pattern							
	SDDE^d,e^	LQIS^d,e^	SSQD^d,e^	CHC^d,e^	SH^d,e^	SSDC^d,e^	SDQS^d,e^	SSDH^d,e^
Locate in Hong Kong^a^	**2.34 (1.15–4.74)**	0.48 (0.25–0.94)	0.48 (0.18–1.29)	**2.23 (1.18–4.21)**	0.74 (0.41–1.35)	0.44 (0.18–1.08)	**2.59 (1.05–6.40)**	1.06 (0.45–2.51)
FD subtype^b^								
Postprandial distress syndrome	0.73 (0.34–1.60)	0.63 (0.29–1.38)	**3.20 (1.07–9.59)**	0.97 (0.51–1.86)	1.86 (0.45–1.66)	2.24 (0.83–6.01)	1.32 (0.53–3.33)	1.06 (0.40–2.82)
Epigastric pain syndrome	1.58 (0.62–4.07)	1.35 (0.52–3.52)	0.21 (0.05–0.92)	1.44 (0.62–3.33)	0.66 (0.27–1.61)	0.65 (0.19–2.17)	0.57 (0.17–1.94)	0.42 (0.11–1.63)
Without IBS diagnosis^c^	0.68 (0.30–1.55)	0.64 (0.29–1.39)	0.91 (0.29–2.81)	0.84 (0.41–1.74)	1.35 (0.67–2.70)	1.25 (0.48–3.24)	0.73 (0.29–1.85)	1.14 (0.47–2.74)
Higher PHQ9 score	1.01 (0.90–1.12)	1.08 (0.98–1.20)	1.08 (0.94–1.25)	1.01 (0.93–1.11)	1.00 (0.92–1.09)	1.06 (0.94–1.20)	0.97 (0.87–1.09)	1.00 (0.89–1.12)
Higher GAD7 score	1.09 (0.98–1.20)	**1.20 (1.08–1.33)**	1.02 (0.88–1.19)	0.92 (0.84–1.00)	1.02 (0.93–1.11)	0.95 (0.85–1.06)	1.03 (0.92–1.16)	0.91 (0.82–1.92)
Higher NDI symptom severity	1.00 (0.99–1.02)	1.00 (0.99–1.02)	1.00 (0.98–1.02)	1.00 (0.99–1.02)	**1.02 (1.01–1.03)**	0.99 (0.97–1.01)	**1.03 (1.01–1.05)**	1.01 (0.99–1.02)
Higher NDI QoL score(s)								
Eating/drinking	1.00 (0.98–1.02)	0.99 (0.98–1.01)	0.97 (0.94–0.99)	1.00 (0.98–1.01)	1.00 (0.99–1.02)	**1.04 (1.01–1.07)**	0.99 (0.97–1.01)	**1.03 (1.01–1.05)**
Sleep disturbance	0.99 (0.97–1.00)	1.00 (0.99–1.02)	0.98 (0.96–1.00)	1.00 (0.99–1.01)	1.00 (0.98–1.01)	1.02 (0.99–1.04)	1.01 (0.99–1.03)	1.01 (0.99–1.03)
Knowledge/control	**1.04 (1.01–1.06)**	0.99 (0.97–1.02)	1.03 (0.99–1.07)	0.99 (0.97–1.01)	0.99 (0.98–1.02)	1.01 (0.98–1.05)	0.97 (0.94–1.00)	0.98 (0.95–1.01)
Interference	1.00 (0.97–1.03)	1.02 (0.99–1.05)	1.00 (0.95–1.04)	0.98 (0.95–1.00)	1.01 (0.99–1.04)	0.96 (0.91–1.00)	1.03 (0.99–1.06)	0.98 (0.95–1.02)

Logistic regression model for each pair of Traditional Chinese Medicine (TCM) diagnostic patterns was adjusted for the remaining TCM diagnostic patterns. Statistically significant results are bolded and underlined

*CHC* Cold-heat complex, *FD* functional dyspepsia, *GAD7* 7-item generalised anxiety disorder scale, *IBS* Irritable bowel syndrome, *LQIS* liver qi invading the stomach, *NDI* Nepean Dyspepsia Index, *PHQ9* 9-item patient’s health questionnaire, *QoL* Quality of life, *SDQS* Spleen deficiency and qi stagnation, *SDDE* Spleen deficiency with dampness encumbrance, *SH* Stomach heat, *SSDC* Spleen-stomach deficiency cold, *SSDH* Spleen-stomach dampness-heat, *SSQD* Spleen-stomach qi deficiency, *TCM* Traditional Chinese Medicine

^**a**^Reference group: Hunan sample

^b^Reference group: Overlapping between postprandial distress syndrome and epigastric pain syndrome

^c^Reference group: With concomitant IBS diagnosis

^d^Results shown in adjusted odds ratios with 95% confidence intervals

^e^*P* values of the Pearson and Deviance tests > 0.10, indicating good model fit

Participants with liver qi invading the stomach were likely to have a higher burden of anxiety symptoms (AOR: 1.20; 95% CI: 1.08–1.33). Regarding the disease-specific quality of life, participants with spleen deficiency and qi stagnation (AOR: 1.03; 95% CI: 1.01–1.05) and stomach heat (AOR: 1.02; 95% CI: 1.01–1.03) tended to have slightly higher NDI symptom severity. Participants with spleen-stomach deficiency cold (AOR: 1.04; 95% CI: 1.01–1.07) and spleen-stomach dampness-heat (AOR: 1.03; 95% CI: 1.01–1.05) tended to have a higher quality of life in the aspects of eating and drinking. Participants with spleen deficiency with dampness encumbrance (AOR: 1.04; 95% CI: 1.01–1.06) were likely to have better knowledge and control over dyspeptic symptoms.

## Discussion

### Standardising traditional Chinese medicine pattern differentiation

In this diagnostic cross-sectional study, we differentiated the TCM diagnostic pattern(s) of 70.7% of the participants in the overall sample (*n* = 400), 73.6% in the Hong Kong sample (*n* = 250), and 64.0% in the Hunan sample (*n* = 150), using respective score-based pattern differentiation rules derived from LTA. With the capability of diagnosing around 70.0% of the participants, we anticipate that these differentiation rules can improve TCM diagnostic reliability in a substantial proportion of FD patients in routine practice. Clinical appropriateness of the diagnostic approach will be judged in consultations, during which the diagnostic decision can be confirmed, adjusted, or rejected via additional information elicited by physical examinations as well as pulse and tongue assessments. To unleash the potential of this approach, patient-reported clinical feature data may be collected online before consultations, and relevant diagnostic results may be made available to TCM practitioners immediately via automated data transmission to electronic health records or if feasible, clinical decision support systems [45]. These are likely to improve the quality of TCM services as limited consultation time can, in turn, be relocated to other key aspects of clinical encounters.

### Traditional Chinese medicine diagnostic patterns and comorbidities

Our results supported the clinical relevance of customising treatments with consideration of individual characteristics, a TCM therapeutic principle that emphasises the significance of taking into account individual differences when tailoring TCM treatments [13, 14].

We demonstrated that participants with the PDS subtype were likely to be diagnosed with the TCM diagnostic patterns of spleen-stomach qi deficiency. In routine practice, TCM practitioners may presume a higher chance of diagnosing spleen-stomach qi deficiency in FD patients with PDS. This positive association may be partly explained by the fact that the dominant symptoms of PDS, namely postprandial fulness and early satiety, are similar to the clinical feature of stomach heaviness (numerical score: 5.5) in the differentiation rule of spleen-stomach qi deficiency.

We also illustrated the association between psychiatric comorbidities and TCM diagnostic patterns. Participants with more severe anxiety symptoms tended to have liver qi invading the stomach. In other words, TCM practitioners may expect a higher possibility of diagnosing liver qi invading the stomach in FD patients with anxiety. In routine practice, TCM practitioners should offer additional psychiatric assessments for patients having this TCM diagnostic pattern and arrange timely referrals if necessary. The association between liver qi invading the stomach and anxiety symptoms seems consistent with TCM theory. Excessive anger, which is likely to be accompanied by anxiety, is thought to cause the stagnation of liver qi [13, 14]. Subsequently, liver qi invading the stomach can occur in accordance with the Five Elements Theory, leading to the co-occurrence of the two conditions [13, 14]. Participants in the overall and Hong Kong samples with liver qi invading the stomach were likely to report the clinical feature of depressed mood, as reflected by its exceptionally high numerical score. However, no significant relationships were identified between that TCM diagnostic pattern and a higher PHQ9 score. It might be due to the fact that PHQ9 measures multiple aspects and consequences of depression, such as fatigue, lack of pleasure in doing daily activities, and suicidal thoughts [40]. In other words, participants who reported having only depressed mood were not necessarily fulfilling multiple criteria listed in PHQ9. In routine practice, TCM practitioners may also consider offering depression assessments to patients with liver qi invading the stomach if deemed clinically appropriate.

### Generalisability of traditional Chinese medicine diagnostic pattern differentiation rules

Our findings revealed the potential necessity for customising treatments with consideration of geographical locations, a TCM theory that highlights the value of factoring in geographical variations when tailoring prescriptions for individual patients [13, 14]. We identified that the distributions of TCM diagnostic patterns of FD differed across the three samples in this study. For instance, the most prevalent TCM diagnostic patterns among participants in the overall, Hong Kong, and Hunan samples were spleen deficiency with dampness encumbrance, cold-heat complex, and liver qi invading the stomach, respectively. Such variation in prevalence might be attributed to the geographical differences between the two regions. According to TCM theory, one’s body constitution and disease progression are influenced by the elements of their surrounding environments, such as season, climate, height above sea level, proximity to mountains and seas, and distribution of natural resources [46, 47]. Other lifestyle factors are also important determinants. These factors include but are not limited to dietary habits, sleep patterns, social interactions, and exercise frequency [46, 47]. Using this framework, we provide a preliminary analysis of how these factors operate differently in the two locations, contributing to the pathogenesis of the most prevalent diagnostic patterns in Hong Kong and Hunan.

The TCM diagnostic pattern of cold-heat complex is commonly caused by a combination of spleen yang deficiency and external dryness and heat [14]. Hong Kong situates in southern China and has a humid and subtropical climate [48]. People in Hong Kong tend to consume cold beverages and desserts to help cope with the heat and dampness, especially during summer. Excess consumption of cold (and raw) food and drinks may gradually damage spleen yang and contribute to spleen yang deficiency [46, 47]. Under the influence of subtropical climate, if those with spleen yang deficiency were also frequent consumers of deep-fried food, a food category deemed dry and heat [46, 47], they were likely to have cold-heat complex in the course of FD [10].

People in China have a relatively long average weekly working time of 46.1 h [49]. Evidence has shown that long working hours and overtime labour are associated with a higher risk of clinical anxiety [50], perhaps due to the lack of leisure time and exercise. In our study, 27.3% of the participants in the Hunan sample were classified as having significant clinical anxiety symptoms that require appropriate follow-up. Therefore, it is expected that liver qi invading the stomach would be prevalent among FD patients in Hunan, given the relationship between this specific TCM diagnostic pattern and anxiety in TCM theory [13, 14].

Furthermore, in terms of numerical scores, the importance of each clinical feature of the same TCM diagnostic patterns varied across samples. For example, the clinical feature of signs and symptoms exacerbated by pressure was the most important in differentiating cold-heat complex for participants in the overall sample and the Hong Kong sample. Nevertheless, it was the second most important clinical feature in the same pattern differentiation rule for the Hunan sample. Similarly, burning sensation in the stomach was an essential clinical feature in differentiating stomach heat among participants in the overall sample and the Hunan sample. However, it was ranked second in the differentiation rule for the same pattern in Hong Kong.

If electronic clinical decision support systems were introduced to TCM practice, local pre-test probabilities of TCM diagnostic patterns of FD could be incorporated into computer-aided TCM diagnostic systems. Together with up-to-date information on the best TCM treatments, such innovation may streamline clinical decision-making by generating patient-specific recommendations based on patients’ clinical information and comorbidities [45], improve the quality of care by allowing accurate diagnoses and appropriate treatments [51], and keep TCM practitioners updated with new evidence on diagnostic methods and treatment strategies [52]. That said, implementation assessments are necessary for evaluating the capacity and preparedness of TCM practitioners and healthcare organisations in adopting these digital health applications.

### Implications for Chinese herbal medicine therapeutic strategies

The variations described above may also influence the choice of appropriate TCM treatments, according to the pattern differentiation principle. Table 4 illustrates the pre-test probabilities of diagnosing different TCM diagnostic patterns among participants in Hong Kong and Hunan, providing hints on a small number of Chinese herbal formulae expected to match these TCM diagnostic patterns. We found that liver qi invading the stomach had the second-highest pre-test probability among all TCM diagnostic patterns in the overall sample. The TCM diagnostic pattern also had the highest pre-test probability in the Hunan sample. These imply that liver qi invading the stomach is likely to be a commonly encountered TCM diagnostic pattern among FD patients in routine practice, at least in one of the geographical locations where our study was conducted. Coincidentally, liver qi invading the stomach is the target TCM diagnostic pattern of the best performing Chinese herbal formulae as indicated in recent network meta-analyses [11, 12]: Xiao Yao Pill, Modified Zhi Zhu Decoction, and Xiao Pi Kuan Wei Decoction.

This coincidence may explain why these Chinese herbal formulae with functions of liver qi soothing and spleen nourishment outperformed the others. In the network meta-analyses, the included randomised controlled trials evaluated the effectiveness of Chinese herbal formulae without considering pattern differentiation for participant recruitment. If a majority of the participants in these trials had liver qi invading the stomach, as suggested by our results, then the use of Xiao Yao Pill, Modified Zhi Zhu Decoction, and Xiao Pi Kuan Wei Decoction would be the most suitable options. If this assumption holds, pattern differentiation may be less important for conditions with a high prevalence of a specific TCM diagnostic pattern because the prescription of specific Chinese herbal formulae targeting that diagnostic pattern would have a high chance of fitting correspondingly. It may explain why some TCM practitioners find pattern differentiation less relevant [53].

### Co-existing traditional Chinese medicine diagnoses among individual patients

Although specific Chinese herbal formulae may be offered as a targeted treatment for FD due to a relatively high prevalence of liver qi invading the stomach, participants with only one TCM diagnostic pattern accounted for only 11.3–14.5% across the three samples. A significant proportion of the participants were diagnosed with more than one TCM diagnostic pattern: 56.2% in the overall sample, 59.6% in the Hong Kong sample, and 52.7% in the Hunan sample.

Via logistic regression analyses, we found that participants in the overall sample with liver qi invading the stomach were likely to experience spleen deficiency and qi stagnation and vice versa. Such co-existence involved as many as 25.5% of the participants in the overall sample. Through targeting the aspect of liver qi invading the stomach, it is expected that Xiao Yao Pill, Modified Zhi Zhu Decoction, and Xiao Pi Kuan Wei Decoction may still partially alleviate symptoms among patients with co-existing spleen deficiency and qi stagnation as they address the same disease mechanism of spleen qi deficiency.

We also revealed other thirteen pairs of co-existing TCM diagnostic patterns from the overall sample dataset. The most common co-existing pair of TCM diagnostic patterns was spleen-stomach qi deficiency and spleen-stomach deficiency cold. It accounted for 32.8% of the participants in the overall sample. The best performing Chinese herbal formulae derived from NMA [11, 12], namely Xiao Yao Pill, Modified Zhi Zhu Decoction, and Xiao Pi Kuan Wei Decoction, do not target spleen-stomach qi deficiency or spleen-stomach deficiency cold. In this case, expertise of TCM practitioners is necessary for judging the severity and prominence of each co-existing pattern, so as to tailor prescriptions accordingly. Such clinical judgements should refer to additional information collected from physical examinations, as well as tongue and pulse assessments.

For example, a patient was judged to have a co-existing spleen-stomach qi deficiency and spleen-stomach deficiency cold under the pattern differentiation rules and was diagnosed with pale tongue with thin and white tongue fur, plus fine or weak pulse [13]. Accordingly, the Chinese herbal formula selected should focus on addressing spleen-stomach qi deficiency, such as Xiang Sha Liu Jun Zi Granules [11], with modifications that address the clinical features manifested from spleen-stomach deficiency cold, such as the addition of Cortex Cinnamomi Radix (or Rou Gui) and Rhizoma Zingiberis (or Gan Jiang) [28]. A combination of Chinese herbal formulae, such as Xiang Sha Liu Jun Zi Granules [11] and Fu Zi Li Zhong Decoction [28], might also be prescribed if clinical features manifested from both TCM diagnostic patterns were prominent.

### Implications for research on the predictability of traditional Chinese medicine diagnostic patterns on treatment response

The application of TCM diagnostic pattern, liver qi invading the stomach, in determining treatment appropriateness resembles the role of predictive biomarkers for personalising oncology treatment. In this case, a single trait (i.e., being diagnosed with liver qi invading the stomach) separates a specific population (i.e., FD patients) with respect to the outcome of interest (i.e., FD symptom alleviation) in response to targeted therapy (i.e., Chinese herbal formulae that soothe liver qi and nourish the spleen) [54, 55]. To answer this research question, future clinical trials may be conducted to assess the potential of liver qi invading the stomach in predicting treatment response in FD patients with different TCM diagnoses [54, 55]. Researchers may consider the following when designing such studies:

*Participant* *eligibility:* The validated Rome IV diagnostic criteria for FD [1] should be adopted as basic eligibility criteria. The pattern differentiation rules derived from our LTA should be used to categorise participants into three subgroups: (i) liver qi invading the stomach-positive, (ii) liver qi invading the stomach-negative, and (iii) those with co-existing liver qi invading the stomach and other TCM diagnostic patterns.

**Fig. 4:**
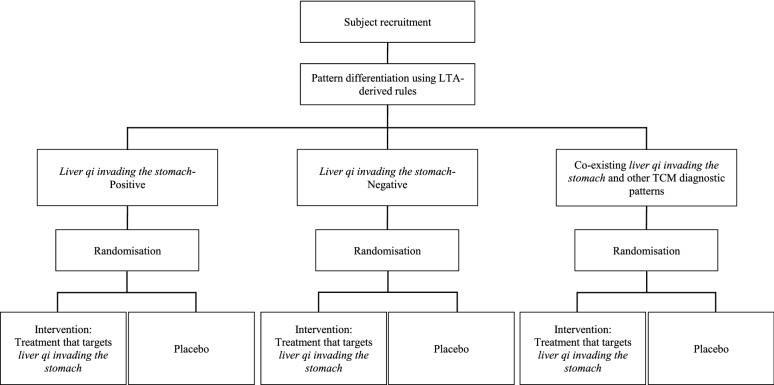
Six-arm trial for comparing the effectiveness of Chinese herbal treatments that target liver qi invading the stomach. *LTA* latent tree analysis, *TCM* traditional Chinese medicine

*Interventions* *and* *comparisons:* A six-arm trial should be performed to compare the effectiveness of specific Chinese herbal formulae that target *liver* *qi* *invading* *the* *stomach* among the subgroups (Fig. 4). In each subgroup, participants are randomly assigned to either the intervention arm or the placebo arm. Xiao Yao Pill, Modified Zhi Zhu Decoction, and Xiao Pi Kuan Wei Decoction are the ideal potential interventions, as shown previously [11, 12]. Compared to the remaining two groups, the effect size among those with co-existing liver qi invading the stomach and other TCM diagnostic patterns may indicate how the presence of additional TCM diagnostic patterns would possibly affect treatment response.

*Outcome* *measures*: Multiple expert-recommended endpoints for FD trials should be selected to facilitate a comprehensive understanding of outcome changes. Global symptom alleviation assessed on a dichotomous scale enables a global assessment [56], while individual symptom alleviation on a seven-point Likert scale assesses the four major symptoms of FD [57], namely postprandial fullness, early satiety, epigastric burning, and epigastric pain. Information on changes in disease-specific quality of life may also be captured using the Nepean Dyspepsia Index [58].

Similar validation trials may be conducted for other conditions to study the possibility of applying LTA-based TCM diagnostic patterns as predictive biomarkers for specific TCM treatments.

### Limitations

Several limitations of this study should be acknowledged. First, since the TCMQ-FD concerns only the clinical features presented in the last two weeks, it may not capture the dynamic property of TCM diagnostic pattern [22]. Therefore, patients should re-attempt the questionnaire immediately prior to follow-up consultations to facilitate accurate diagnoses. Second, although pulse and tongue features are pivotal for TCM diagnosis, we did not include them in the LTA due to the absence of promising automated diagnostic apparatuses that can obtain relevant data in an objective manner. Third, the pattern differentiation rules established using data collected from the Hong Kong and Hunan samples may not be generalised to populations outside the two regions. It is because the elements related to their surrounding environments and lifestyle, such as season, climate, diet, and stress level, may not be the same as FD populations in other regions. These elements are expected to influence the prevalence of different TCM diagnostic patterns and their constituting clinical features according to TCM theory [46, 47]. Fourth, since climate may influence the distribution of TCM diagnostic patterns in a geographical region [13, 14], results might have been different if this cross-sectional study were conducted in summer when dampness is the dominant qi of the season [14].

## Conclusions

We derived eight LTA-based TCM diagnostic patterns of FD using data collected from Hong Kong and Hunan and established relevant score-based pattern differentiation rules for routine practice. We revealed the associations between FD subtypes, as well as psychiatric comorbidities, and TCM diagnostic patterns of FD. Variations in the prevalence of TCM diagnostic patterns and constituting clinical features among FD patients residing in different geographical locations were also quantified. Future updates of the ICD-11, TCM textbooks, and clinical guidelines on FD should stress the importance of considering individual characteristics and geographical locations in routine practice. Suppose clinical decision support systems were introduced to TCM practice. In this case, the results of pattern differentiation of FD patients could be made available to TCM practitioners via automated data transmission to electronic health record systems, along with local pre-test probabilities of TCM diagnostic patterns of FD and recommendations for the corresponding TCM treatments. This innovation could enable the implementation of accurate diagnoses and appropriate treatments. That said, implementation assessments are recommended for evaluating the facilities' capacity and preparedness and TCM practitioners' adoption of digital health applications.

## Supplementary Information

**Additional file 1: Appendix 1.** Definitions of key terms used in this study. **Appendix 2.** Probabilistic co-occurring clinical features in the latent variables of the global latent tree model illustrated in Fig. 2.

## Data Availability

The datasets used and/or analysed during the current study are available from the corresponding author upon reasonable request.

## Abbreviations

AOR: Adjusted odds ratio
CI: Confidence interval
EPS: Epigastric pain syndrome
FD: Functional dyspepsia
GAD7: General anxiety disorder-7
H. pylori: Helicobacter pylori
IBS: Irritable bowel syndrome
LTA: Latent tree analysis
LTM: Latent tree models
NDI: Nepean Dyspepsia Index
PDS: Postprandial distress syndrome
PHQ9: Patient Health Questionnaire-9
TCM: Traditional Chinese Medicine
TCMQ-FD: Traditional Chinese Medicine Clinical Feature Questionnaire for Functional Dyspepsia
