# Supplementary material for: Quantification of prevalence, clinical characteristics, co-existence, and geographic variations of traditional Chinese medicine diagnostic patterns via latent tree analysis-based differentiation rules among functional dyspepsia patients
Source: Chin Med. 2022 Aug 30;17:101. doi: 10.1186/s13020-022-00656-x (PMC9425972; doi:10.1186/s13020-022-00656-x)
Supplement: Supplementary file 1 — Additional file 1: Appendix 1. Definitions of key terms used in this study. Appendix 2. Probabilistic co-occurring clinical features in the latent variables of the global latent tree model illustrated in Fig. 2. [file 13020_2022_656_MOESM1_ESM.docx]

**Appendix 1.** Definitions of key terms used in this study

| **Key term** | **Definition** |
| --- | --- |
| Bayesian information criterion | A parameter that reflects both the fitting and complexity of a latent tree model:   - The lower the better when it takes a positive value - The higher the better when it takes a negative value |
| Dataset for Hong Kong sample | The data collected from the 250 participants in Hong Kong |
| Dataset for Hunan sample | The data collected from the 150 participants in Hunan |
| Dataset for overall sample | The data collected from all 400 participants (250 in Hong Kong and 150 in Hunan) |
| Hong Kong sample | Hong Kong participants only (*n* = 250) |
| Hunan sample | Hunan participants only (*n* = 150) |
| *Lantern* | A freely available software developed to facilitate latent tree analysis on personal computers. It explores the best latent tree model that fits categorical datasets by adopting one of the following algorithms:   - Bridged-islands – suggested for datasets with hundreds to one thousand observed variables - Extension adjustment simplification until termination – suggested for datasets with dozens to one hundred observed variables   It also acquires the maximum likelihood estimates of the probabilistic parameters of a latent tree model with the expectation-maximisation algorithm |
| Latent tree analysis | A statistical method that discovers the best latent tree model that fits the data and helps derive pattern differentiation rules from cluster analysis of the model |
| Latent tree model | A statistical model for cluster analysis of categorical data. It shows the relationships between observed variables (*i.e.*, patient-reported clinical features) and latent variables in qualitative and quantitative perspectives:   - Qualitatively, it is an undirected tree-like structure with the observed variables located at the leaf nodes and the latent variables at the internal nodes - Quantitatively, it explains the relationships between observed variables and their latent variables using conditional probability distributions |
| Overall sample | Hong Kong participants (*n* = 250) and Hunan participants (*n* = 150) |
| Participants | Participants who participated in this study |
| Patients/FD patients | Patients with functional dyspepsia (FD) around the world |
| Samples | Different groups of participants in this study |
| Soft label | A cluster label that is assigned to the participant using the soft clustering approach. The label represents the probability of that participant belonging to the cluster (*i.e.*, Traditional Chinese Medicine (TCM) diagnostic pattern) |
| *Traditional Chinese Medicine Clinical Feature Questionnaire for Functional Dyspepsia* | A fifty-five-item questionnaire that guides the collection of self-reported clinical feature data of FD. Participants are to rate the severity of each clinical feature on a five-point Likert scale, with a higher numerical rating indicating higher severity  The items were developed from two sources: (i) a systematic review of TCM diagnostic instruments for FD ^[1]^ and (ii) the *2017 Chinese Medicine Expert Consensus on Functional Dyspepsia Diagnosis in China* ^[2]^ |

^[1]^Ho L, Chung VC, Wong CH, Wu IX, Lan KC, Wu D, et al. Evaluating Traditional Chinese Medicine Diagnostic Instruments for Functional Dyspepsia: Systematic Review on Measurement Properties. *Integr Med Res*. 2021;10(3):100713

^[2]^Study group of Gastrointestinal Motility - Chinese Society of Gastroenterology, Study group of Functional Gastrointestinal Disorders - Chinese Society of Gastroenterology. Chinese Expert Consensus on Functional Dyspepsia Management (2015) (Chinese). *Chin J Dig*. 2015;36(4):217-28

**Appendix 2.** Probabilistic co-occurring clinical features in the latent variables of the global latent tree model illustrated in Figure 2

| **Latent variable *Y*0** | | |
| --- | --- | --- |
| *Clinical feature* | *Participants classified into Y0* | *Participants not classified into Y0* |
| Oppression in the chest | 0.65 | 0.03 |
| Stomach heaviness | 0.30 | 0.02 |
| Weight loss | 0.24 | 0.03 |
| **Latent variable *Y*1** | | |
| *Clinical feature* | *Participants classified into Y1* | *Participants not classified into Y1* |
| Borborygmus | 0.40 | 0.07 |
| Yellowish urine | 0.39 | 0.07 |
| Belching | 0.63 | 0.33 |
| **Latent variable *Y*2** | | |
| *Clinical feature* | *Participants classified into Y2* | *Participants not classified into Y2* |
| Passing stools with difficulty | 0.76 | 0.01 |
| Tenesmus | 0.70 | 0.03 |
| **Latent variable *Y*3** | | |
| *Clinical feature* | *Participants classified into Y3* | *Participants not classified into Y3* |
| Excessive phlegm or salivation | 0.57 | 0.02 |
| Foreign body sensation in the throat | 0.62 | 0.08 |
| **Latent variable *Y*4** | | |
| *Clinical feature* | *Participants classified into Y4* | *Participants not classified into Y4* |
| Foul-smelling stools | 0.51 | 0.03 |
| Hard stools followed by soft stools | 0.52 | 0.04 |
| Undigested food in stools | 0.31 | 0.02 |
| **Latent variable *Y*5** | | |
| *Clinical feature* | *Participants classified into Y5* | *Participants not classified into Y5* |
| Diarrhoea | 0.73 | 0.06 |
| Unformed stools | 0.53 | 0.03 |
| **Latent variable *Y*6** | | |
| *Clinical feature* | *Participants classified into Y6* | *Participants not classified into Y6* |
| Swift digestion with rapid hungering | 0.76 | 0.12 |
| Torpid intake | 0.50 | 0.18 |
| Thirst without desire to drink | 0.30 | 0.08 |
| **Latent variable *Y*7** | | |
| *Clinical feature* | *Participants classified into Y7* | *Participants not classified into Y7* |
| Aversion to cold | 0.94 | 0.08 |
| Cold hands and feet | 0.76 | 0.12 |
| **Latent variable *Y*8** | | |
| *Clinical feature* | *Participants classified into Y8* | *Participants not classified into Y8* |
| Sallow complexion | 0.36 | 0.02 |
| Poor sleep quality | 0.61 | 0.31 |
| **Latent variable *Y*9** | | |
| *Clinical feature* | *Participants classified into Y9* | *Participants not classified into Y9* |
| Lassitude of spirit | 0.94 | 0.21 |
| Reluctance to speak | 0.58 | 0.00 |
| Lack of strength | 0.64 | 0.02 |
| **Latent variable *Y*10** | | |
| *Clinical feature* | *Participants classified into Y10* | *Participants not classified into Y10* |
| Heavy-headedness | 0.55 | 0.01 |
| Body heaviness | 0.58 | 0.03 |
| Dizziness | 0.51 | 0.03 |
| **Latent variable *Y*11** | | |
| *Clinical feature* | *Participants classified into Y11* | *Participants not classified into Y11* |
| Depressed mood | 0.75 | 0.02 |
| Irritability | 0.72 | 0.08 |
| **Latent variable *Y*12** | | |
| *Clinical feature* | *Participants classified into Y12* | *Participants not classified into Y12* |
| Acid vomiting | 0.53 | 0.02 |
| Hiccup | 0.61 | 0.11 |
| Vomiting and nausea | 0.40 | 0.04 |
| **Latent variable *Y*13** | | |
| *Clinical feature* | *Participants classified into Y13* | *Participants not classified into Y13* |
| Bland taste in the mouth | 0.45 | 0.00 |
| Dry mouth | 0.72 | 0.18 |
| Bitter taste in the mouth | 0.43 | 0.04 |
| Fetid mouth odour | 0.41 | 0.08 |
| **Latent variable *Y*14** | | |
| *Clinical feature* | *Participants classified into Y14* | *Participants not classified into Y14* |
| Signs and symptoms exacerbated by mood | 0.62 | 0.06 |
| Signs and symptoms exacerbated by cold | 0.58 | 0.17 |
| Signs and symptoms relieved by pressure | 0.28 | 0.05 |
| **Latent variable *Y*15** | | |
| *Clinical feature* | *Participants classified into Y15* | *Participants not classified into Y15* |
| Distension and fullness in the stomach | 0.82 | 0.18 |
| Dull pain in the stomach | 0.56 | 0.04 |
| Gastric upset | 0.59 | 0.06 |
| Signs and symptoms exacerbated by pressure | 0.47 | 0.05 |
| Signs and symptoms exacerbated by ingestion | 0.49 | 0.07 |

*Note: Each latent variable is divided into two possible clusters of participants. Based on the manifestation of probabilistic co-occurring clinical features, one of the clusters included participants that were classified into that latent aspect, while the other included those that were not classified into that latent aspect.*
